# In situ adaptation and ecological release facilitate the occupied niche expansion of a non‐native Madagascan day gecko in Florida

**DOI:** 10.1002/ece3.7749

**Published:** 2021-06-23

**Authors:** Thomas W. Fieldsend, Nicolas Dubos, Kenneth L. Krysko, Christopher J. Raxworthy, Sparkle L. Malone

**Affiliations:** ^1^ Department of Biological Sciences Florida International University Miami FL USA; ^2^ Département Écologie et Gestion de la Biodiversité Muséum National d'Histoire Naturelle Paris France; ^3^ Division of Herpetology Florida Museum of Natural History Gainesville FL USA; ^4^ Division of Vertebrate Zoology Department of Herpetology American Museum of Natural History New York City NY USA

**Keywords:** competitive exclusion, ecological niche modeling, fundamental niche, herpetofauna, non‐native species, *Phelsuma grandis*, *Phelsuma kochi*, realized niche, reptiles, species distribution modeling

## Abstract

**Aim:**

To investigate whether the frequently advocated climate‐matching species distribution modeling approach could predict the well‐characterized colonization of Florida by the Madagascar giant day gecko *Phelsuma grandis*.

**Location:**

Madagascar and Florida, USA.

**Methods:**

To determine the climatic conditions associated with the native range of *P*. *grandis*, we used native‐range presence‐only records and *Bioclim* climatic data to build a Maxent species distribution model and projected the climatic thresholds of the native range onto Florida. We then built an analogous model using Florida presence‐only data and projected it onto Madagascar. We constructed a third model using native‐range presences for both *P*. *grandis* and the closely related parapatric species *P*. *kochi*.

**Results:**

Despite performing well within the native range, our Madagascar *Bioclim* model failed to identify suitable climatic habitat currently occupied by *P*. *grandis* in Florida. The model constructed using Florida presences also failed to reflect the distribution in Madagascar by overpredicting distribution, especially in western areas occupied by *P*. *kochi*. The model built using the combined *P*. *kochi*/*P*. *grandis* dataset modestly improved the prediction of the range of *P*. *grandis* in Florida, thereby implying competitive exclusion of *P*. *grandis* by *P*. *kochi* from habitat within the former's fundamental niche. These findings thus suggest ecological release of *P*. *grandis* in Florida. However, because ecological release cannot fully explain the divergent occupied niches of *P*. *grandis* in Madagascar versus Florida, our findings also demonstrate some degree of in situ adaptation in Florida.

**Main conclusions:**

Our models suggest that the discrepancy between the predicted and observed range of *P*. *grandis* in Florida is attributable to either in situ adaptation by *P*. *grandis* within Florida, or a combination of such in situ adaptation *and* competition with *P*. *kochi* in Madagascar. Our study demonstrates that climate‐matching species distribution models can severely underpredict the establishment risk posed by non‐native herpetofauna.

## Graphical abstract

Climate‐matching species distribution models (SDMs) are frequently used to predict biotic invasions. Our manuscript details how both release from native‐range competitors and in situ adaptation within the non‐native range have enabled the day gecko *Phelsuma grandis* to flourish in Florida under bioclimatic conditions classified as highly unsuitable by an otherwise‐well‐performing climate‐matching SDM. By identifying the large discrepancy between the predicted and observed range of *P. grandis* in Florida, our study highlights the shortcomings of climate‐matching SDMs as conservation management tools, while also providing valuable empirical evidence of the ecological and evolutionary processes underpinning this discrepancy.

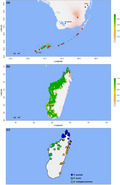

## 1 INTRODUCTION

Species distribution models (SDMs)—sometimes referred to as environmental or ecological niche models (ENMs)—combine taxon‐specific distributional data with ecologically relevant data to estimate the likelihood of potential or actual occurrence of the taxon of interest at spatiotemporal locations for which reliable occurrence data are unavailable (Elith & Leathwick, 2009; Uden et al., 2015). Correlative “climate‐matching” SDMs use the bioclimatic characteristics of a taxon's observed range to identify regions of potential bioclimatic suitability outside of its known range (Engeman et al., 2011; Hattab et al., 2017; Uden et al., 2015) and are considered a useful tool for the management of non‐native herpetofauna (reptiles and amphibians) (Bomford et al., 2009; Fujisaki et al., 2009; van Wilgen et al., 2009).

Maxent (Phillips et al., 2006) is one of the most popular methods for modeling species distributions (Merow et al., 2013) and is widely used in the study of non‐native reptiles (Angetter et al., 2011; Buckland et al., 2014; Cohen, 2017; Dowell et al., 2016; Falcón et al., 2012; Jarnevich et al., 2018; Mothes et al., 2019; Mutascio et al., 2018; Nania et al., 2020; Pyron et al., 2008; Rödder et al., 2008; Weterings & Vetter, 2018). Maxent has been shown to generally outperform equivalent methods (Elith et al., 2006; Gogol‐Prokurat, 2011), returning highly accurate predictions even with small sets of presence‐only data (Gogol‐Prokurat, 2011; Merow et al., 2013; Pearson et al., 2007).

Despite their widespread use, some species distribution modeling approaches have been criticized for their “ecologically untenable” assumptions (Dormann, 2007:387) and inability to capture and characterize environmental heterogeneity at biologically relevant spatial scales (Sears & Angilletta, 2015). Also problematic is the fact that SDMs are often constructed using observed realized niche data (Pearson & Dawson, 2003; Veloz et al., 2012), when in fact the focal taxon's fundamental niche may be significantly larger, but constrained by factors including dispersal limitations and biotic interactions (Boulangeat et al., 2012; Li et al., 2014; Pearson & Dawson, 2003; Rodriguez‐Cabal et al., 2012). In addition, SDM predictions can vary dramatically according to the data and assumptions on which they are built (e.g., Anderson & Raza, 2010; Dowell et al., 2016; Pearson et al., 2006; Pyron et al., 2008; Radosavljevic & Anderson, 2014), leading to uncertainty when interpreting their outputs. Considerable shortcomings such as these have led some to conclude that climate‐matching SDMs may not be warranted as a risk assessment tool for non‐native herpetofauna (Li et al., 2014).

Florida is home to more established non‐native species of reptile and amphibian than anywhere else on Earth (Krysko et al., 2016), and SDMs based wholly or partly on climate‐matching techniques have been developed for a wide range of non‐native herpetofauna in the state (e.g., Mothes et al., 2019), including the Burmese python (*Python bivittatus*) (Pyron et al., 2008; Rodda et al., 2009), Argentine black‐and‐white tegu (*Salvator merianae*) (Jarnevich et al., 2018), Nile monitor (*Varanus niloticus*) (Cohen, 2017; Dowell et al., 2016), and green iguana (*Iguana iguana*) (Falcón et al., 2012), all of which are considered to be problematic invasive species.

**FIGURE 1:**
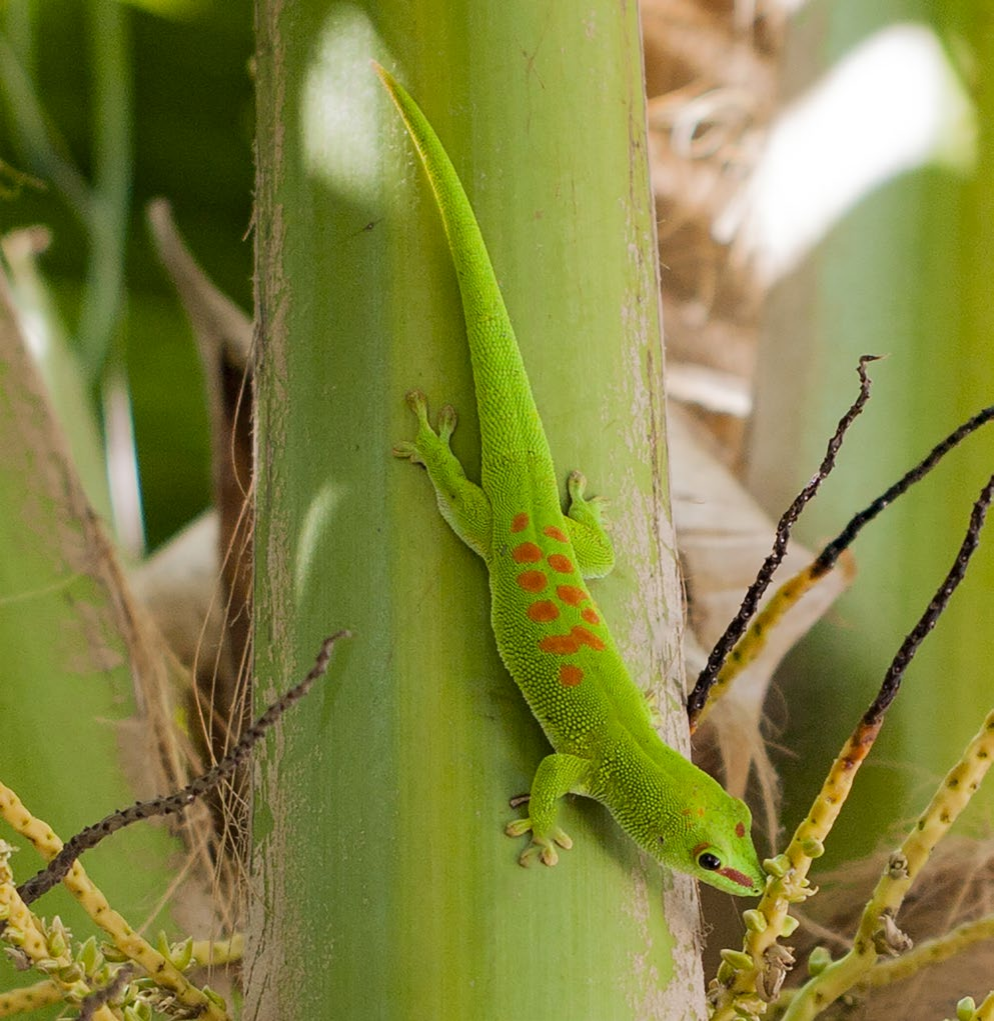
Non‐native Madagascar giant day gecko (*Phelsuma grandis*) in situ on a non‐native coconut palm (*Cocos nucifera*) on Grassy Key, Monroe County, Florida, USA. Photograph (UF‐Herpetology photographic voucher 170124) by Kenneth L. Krysko

In this study, we tested the predictive accuracy of the climate‐matching species distribution modeling approach using range data for the Madagascar giant day gecko *Phelsuma grandis* Gray 1870 (Figure 1). In both its native and colonized range, *P*. *grandis* can be found in a variety of habitat types, including primary forests, orchards, highly degraded forests, and anthropogenic habitats (Blumgart et al., 2017; D'Cruze & Kumar, 2011; D'Cruze et al., 2009; Dubos et al., 2014; Krysko et al., 2019; Krysko et al., 2003; Raselimanana et al., 2000; Raxworthy & Nussbaum, 1994); we therefore expected that habitat variables would be poor predictors of *P*. *grandis* occupancy and thus concluded that a climate‐matching SDM approach was desirable. Using native‐range presence‐only data, we generated a predicted distribution for *P*. *grandis* in Florida—a region in which the species is well established and its range well documented (Fieldsend & Krysko, 2019b)—which we then compared with the observed distribution. We also built an analogous model using Florida *P*. *grandis* presence‐only data, which we projected onto both Florida and Madagascar, allowing us to check the degree of agreement between the outputs of the two models. Finally, we built a model combining native‐range presence data for *P*. *grandis* and the parapatric (Raxworthy et al., 2007), closely related species *P*. *kochi* Mertens 1954, to test whether our climate‐matching SDM approach provided evidence for the competitive exclusion of *P*. *grandis* from a portion of its fundamental niche by *P*. *kochi*.

## 2 METHODS

We compiled a dataset of 71 georeferenced native‐range *Phelsuma grandis* presence points (Appendix S1). Only confirmed observations from peer‐reviewed literature were included in the dataset. Points were checked against the species’ known native distribution in northern Madagascar (Raxworthy et al., 2007) to confirm their accuracy. Global Biodiversity Information Center (GBIF) data are often used in the construction of SDMs (e.g., Mothes et al., 2019; Nania et al., 2020; Suzuki‐Ohno et al., 2017; Weterings & Vetter, 2018), but were not included in this list of presences as they include iNaturalist “Research Grade” observations (Boone & Basille, 2019) of *P*. *grandis*, many of which are actually misidentifications of *P. kochi* or *P*. *madagascariensis* Gray 1831 (pers. obs.). A preliminary projection of the 71 presence points onto the native range showed that nine were located fractionally offshore due to either recording or projection errors, and exacerbated by the partially coastal distribution of the species (see Appendix S1), leaving a total of 62 presence records, well above the minimum number required to develop an adequate Maxent model (Pearson et al., 2007; van Proosdij et al., 2016).

We collated 239 georeferenced observations of *P*. *grandis* from southern Florida from the Florida Museum of Natural History's Division of Herpetology records and from verified personal observations by the authors (Appendix S2). Two data points were removed as they were known to represent either singleton records or now‐extirpated populations. Duplicate coordinates were then also removed, resulting in 115 unique records. Due to the coarse resolution of the spatial data relative to the small size of some of the Florida Keys, only 70 of these 115 records were categorized as being on land, with the remaining 45 points being omitted from the final dataset.

We combined twenty‐one georeferenced native‐range *P*. *kochi* observations taken from the Supplementary data of Raxworthy et al. (2007) with the aforementioned native‐range *P*. *grandis* presence records to produce a *P*. *kochi*/*P*. *grandis* dataset (Appendix S3).

Nineteen *Bioclim* variables were downloaded for both Madagascar and Florida from the WorldClim database (http://www.worldclim.org/) (Hijmans et al., 2005) at 30 arc‐second resolution (~1 km^2^) for use as predictor variables in the models. *Bioclim* variables were selected as they are the most commonly used environmental variables in species distribution modeling (Booth et al., 2014), thus making them the ideal data with which to test the validity of the climate‐matching SDM approach. A detailed explanation of the creation and interpretation of these variables is given in O’Donnell and Ignizio (2012).

We used the Madagascar *P. grandis* presence records and *Bioclim* variables to develop a *P*. *grandis* native‐range SDM (the “Madagascar model”) trained on the whole of Madagascar using the Maxent algorithm (Phillips et al., 2006) via the “dismo” package (Hijmans et al., 2017) in R version 3.5.3 (R Core Team, 2013). The assumptions of the Maxent algorithm are discussed in great detail elsewhere (Elith et al., 2011; Merow et al., 2013). The model incorporated a target‐group background (Phillips et al., 2009) consisting of 21,111 georeferenced Madagascar presence records for the Order Squamata, as downloaded from GBIF (09 March 2021) (Appendix S4).

Model pre‐evaluation included fivefold cross‐validation, executed using the *ENMevaluate* function in “ENMeval,” which also incorporates the Maxent algorithm (Muscarella et al., 2014). The regularization multiplier was set to 3 to reduce the risk of overfitting and smooth model output (Elith et al., 2011; Merow et al., 2013; Mutascio et al., 2018; Radosavljevic & Anderson, 2014); all other model parameters were run as default, with all Maxent feature classes allowed. The optimum Maxent feature class/class combination was determined to be that which returned the lowest average AUC_DIFF_ (a measure of model overfitting; see Warren & Seifert, 2011) while also having an associated training AUC ≥ 0.9 (thus indicating excellent model performance; Swets, 1988). Thereafter, presence records were randomly partitioned 2:1 for use as training and validation datasets, respectively, with two thirds of the data used to build the model proper using the parameters described above, and the remaining third withheld to assess the model performance. The model proper was projected onto Florida and the Caribbean to determine which areas would be deemed bioclimatically suitable for *P*. *grandis*, and was validated using AUC and AUC_DIFF_ (i.e., AUC_TRAIN_–AUC_TEST_; Muscarella et al., 2014).

The process of creation, projection, and analysis of a second, combined‐species “*kochi*/*grandis* model” was identical to that of the Madagascar model, except that *P*. *grandis*‐only presence data were substituted with the *P*. *kochi*/*P*. *grandis* combined dataset previously described. Similarly, a third “Florida model” was pre‐evaluated, trained, and validated using presence/background data for the whole of Florida—with a target‐group background generated using 26,037 georeferenced Florida presence records for the Order Squamata from the Florida Museum of Natural History's Division of Herpetology records (09 March 2021) (Appendix S5)—instead of Madagascar, but was otherwise identical in construction. We projected the Florida model onto Florida to assess its predictive performance in the invasive range, and also projected it onto Madagascar to assess its ability to predict the native range.

## 3 RESULTS

**FIGURE 2:**
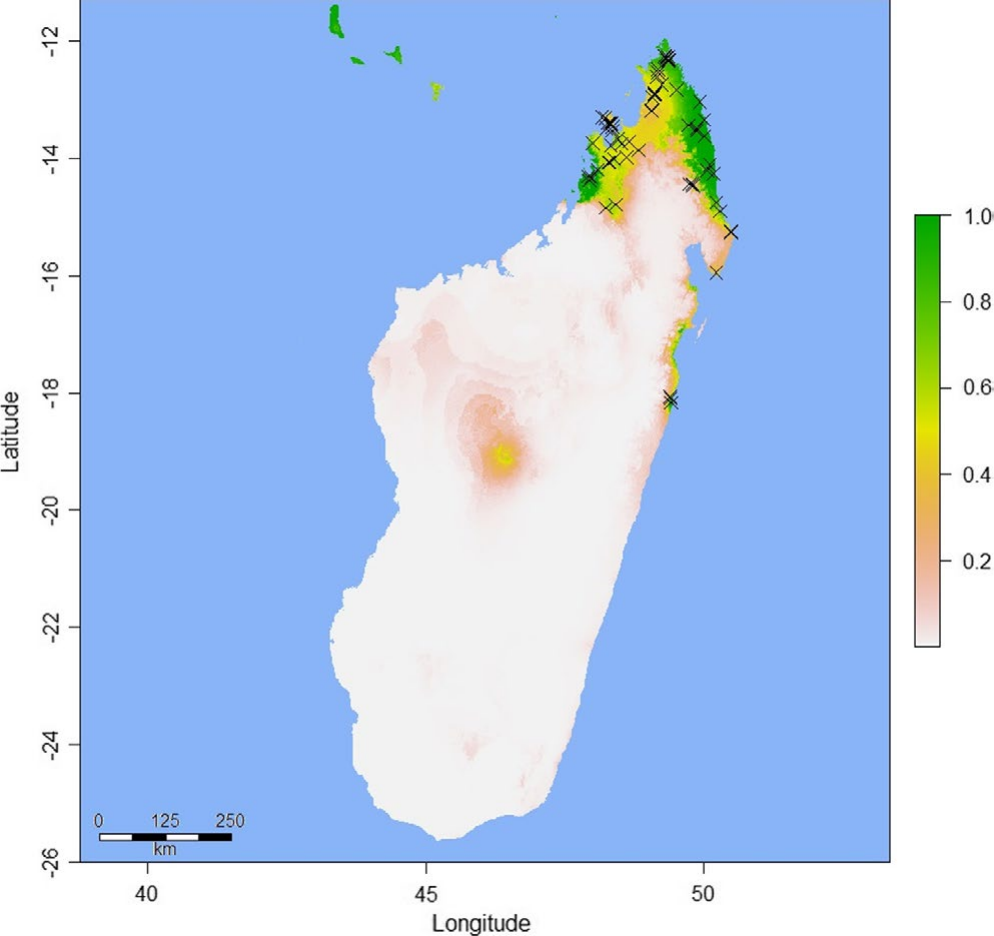
“Madagascar” Maxent model showing predicted habitat suitability for *Phelsuma grandis* in its native range of Madagascar. Crosses denote the georeferenced *P*. *grandis* observations (*n* = 62) used to construct the model. The scale bar to the right indicates the degree of predicted habitat suitability, with higher scores representing predicted higher suitability, with the range of possible values 0–1

**FIGURE 3:**
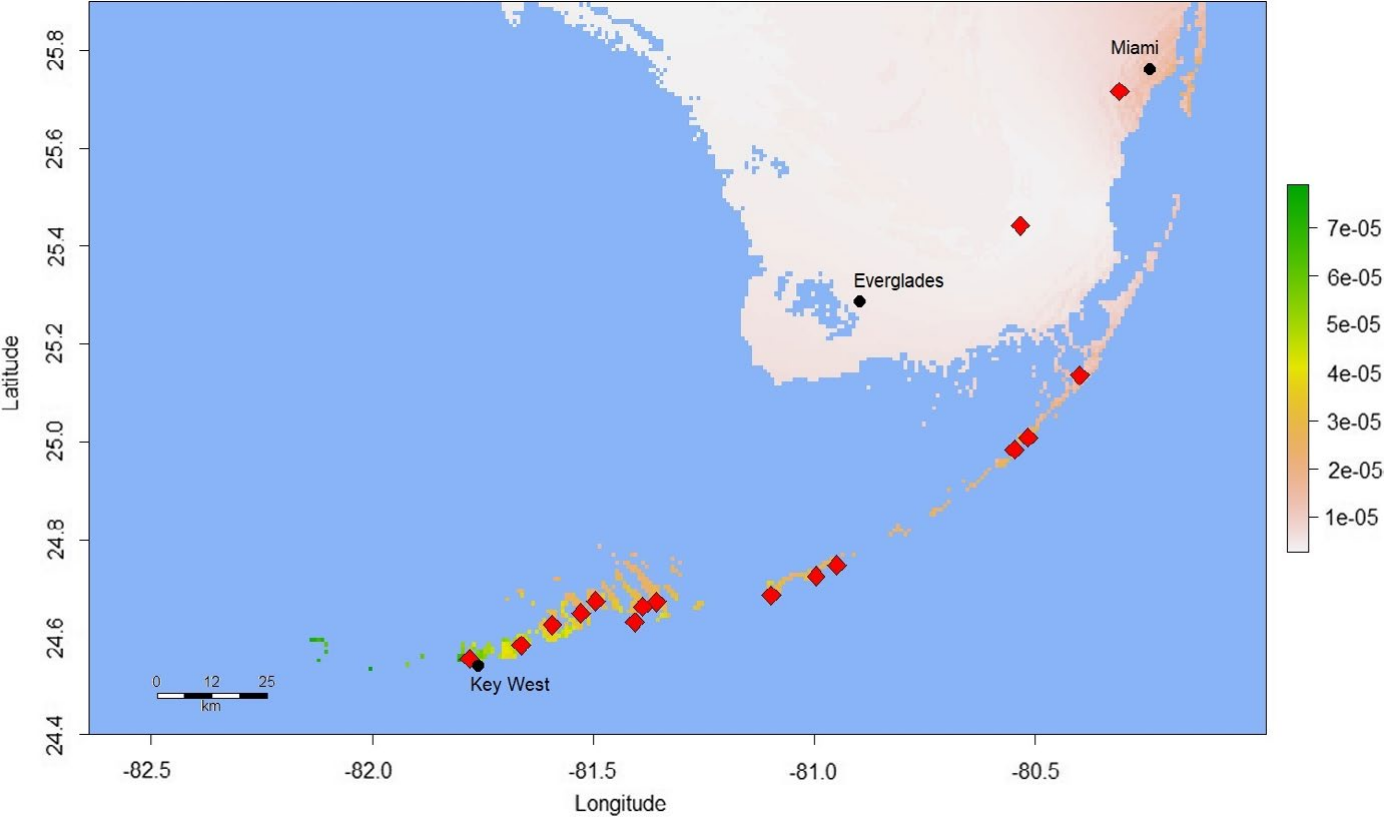
“Madagascar” Maxent model showing predicted habitat suitability for *Phelsuma grandis* in southern Florida, where the species was introduced in the 1990s and is now widely established. Diamonds denote the approximate locations of known *P*. *grandis* populations. The scale bar to the right indicates the degree of predicted habitat suitability, with higher scores representing predicted higher suitability, with the range of possible values 0–1

**FIGURE 4:**
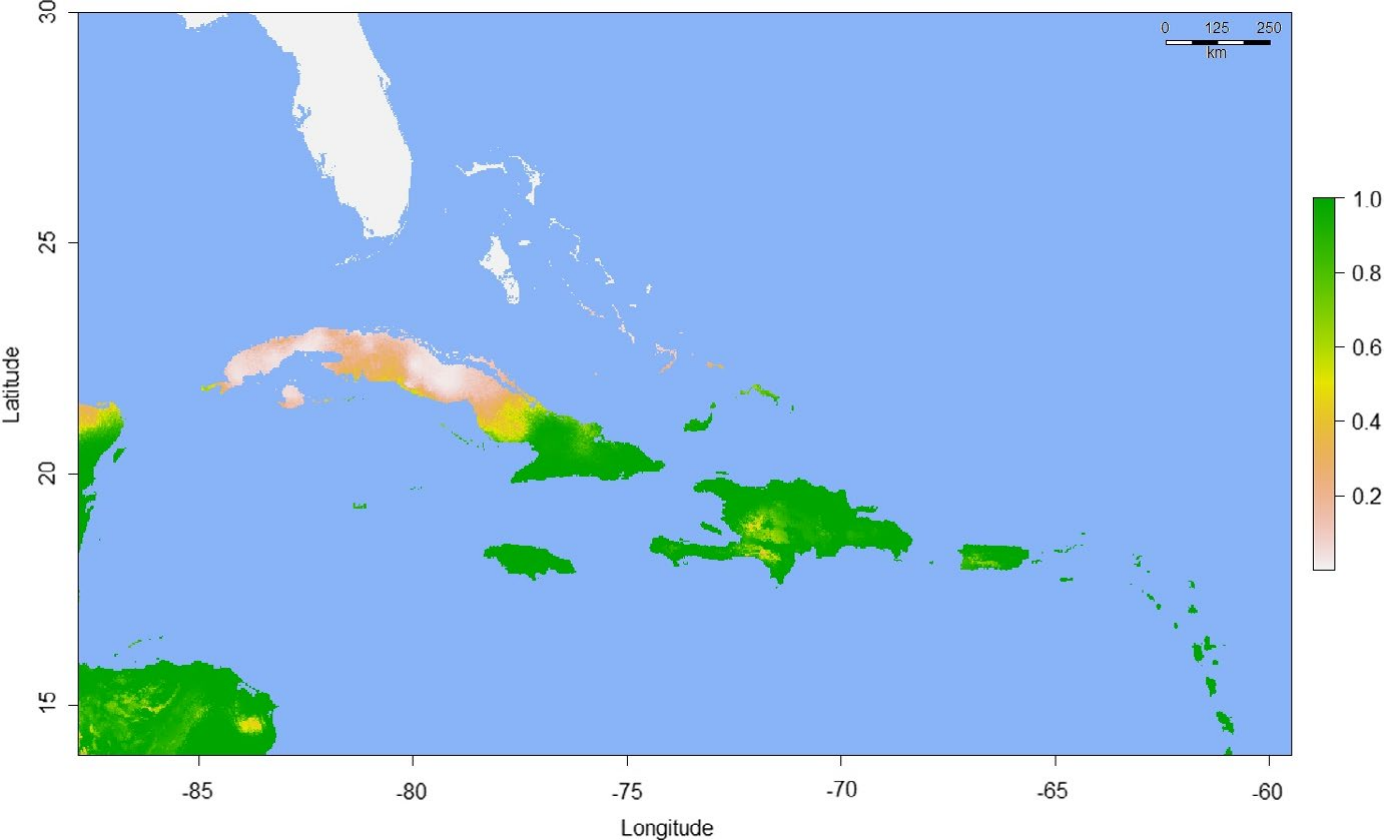
“Madagascar” Maxent model showing predicted habitat suitability for *Phelsuma grandis* in Florida and the Caribbean. The scale bar to the right indicates the degree of predicted habitat suitability, with higher scores representing predicted higher suitability, with the range of possible values 0–1

Model parameters and performance statistics are summarized in Table 1. Fivefold cross‐validation in “ENMeval” returned training AUC values ≥0.9193 for all three models—indicating very high predictive performance (Swets, 1988)—and low average AUC_DIFF_ (≤0.0206) in all cases, confirming that overfitting was not occurring (Warren & Seifert, 2011). AUC and AUC_DIFF_ results were similar for the models proper (AUC ≥ 0.939; AUC_DIFF_ ≤ 0.014), again indicating satisfactory performance. Visual inspection of the Madagascar model projection (Figure 2) confirms that areas of predicted bioclimatic suitability closely match the known native range (Raxworthy et al., 2007). Predicted suitability values for the validation data points ranged from 0.233 to 0.999 (mean = 0.635; median = 0.505). Two bioclimatic variables were responsible for 88.2% of the permutation importance of the Madagascar model: Temperature Seasonality (BIO4; 70.8%) and Precipitation of the Driest Month (BIO14; 17.4%). When projected onto Florida (Figure 3), the Madagascar model identified no areas of bioclimatic suitability, although we observed a general trend of higher suitability for more southerly tropical and subtropical areas, especially the Florida Keys. Predicted suitability values for the 70 Florida *P. grandis* locations ranged from 0.000003 to 0.00008 (mean = 0.00003), with a possible range of values from 0 (highly unsuitable habitat) and 1 (perfectly suitable habitat). The 10th percentile presence threshold (P10) (Cao et al., 2013; Pearson et al., 2007; Phillips et al., 2006) for this model (0.415) yielded an omission error rate of 100% when applied to the Florida *P*. *grandis* presence records. A wider projection covering much of the Caribbean (Figure 4) identified abundant suitable habitat for *P*. *grandis* in the more tropical region, providing further evidence that the lack of suitable habitat identified in Florida is not simply an artifact of an overfitted model.

**TABLE 1:** Parameters and performance statistics of the Maxent species distribution models used in this study

Model	Number of presence points	Number of background points	Regularization multiplier	Selected Maxent feature class combination	Training AUC (fivefold cross‐validation)	Average AUC_DIFF_ (fivefold cross‐validation)	AUC (model proper)	AUC_DIFF_ (model proper)	P10 (model proper)
Madagascar	62	21,111	3	LQ	0.9391	0.0061	0.966	0.0121	0.415
Florida	70	26,037	3	LQHPT	0.9781	0.0069	0.982	−0.0005	0.138
*kochi*/*grandis*	93	21,111	3	LQHP	0.9193	0.0206	0.939	0.0140	0.427

**FIGURE 5:**
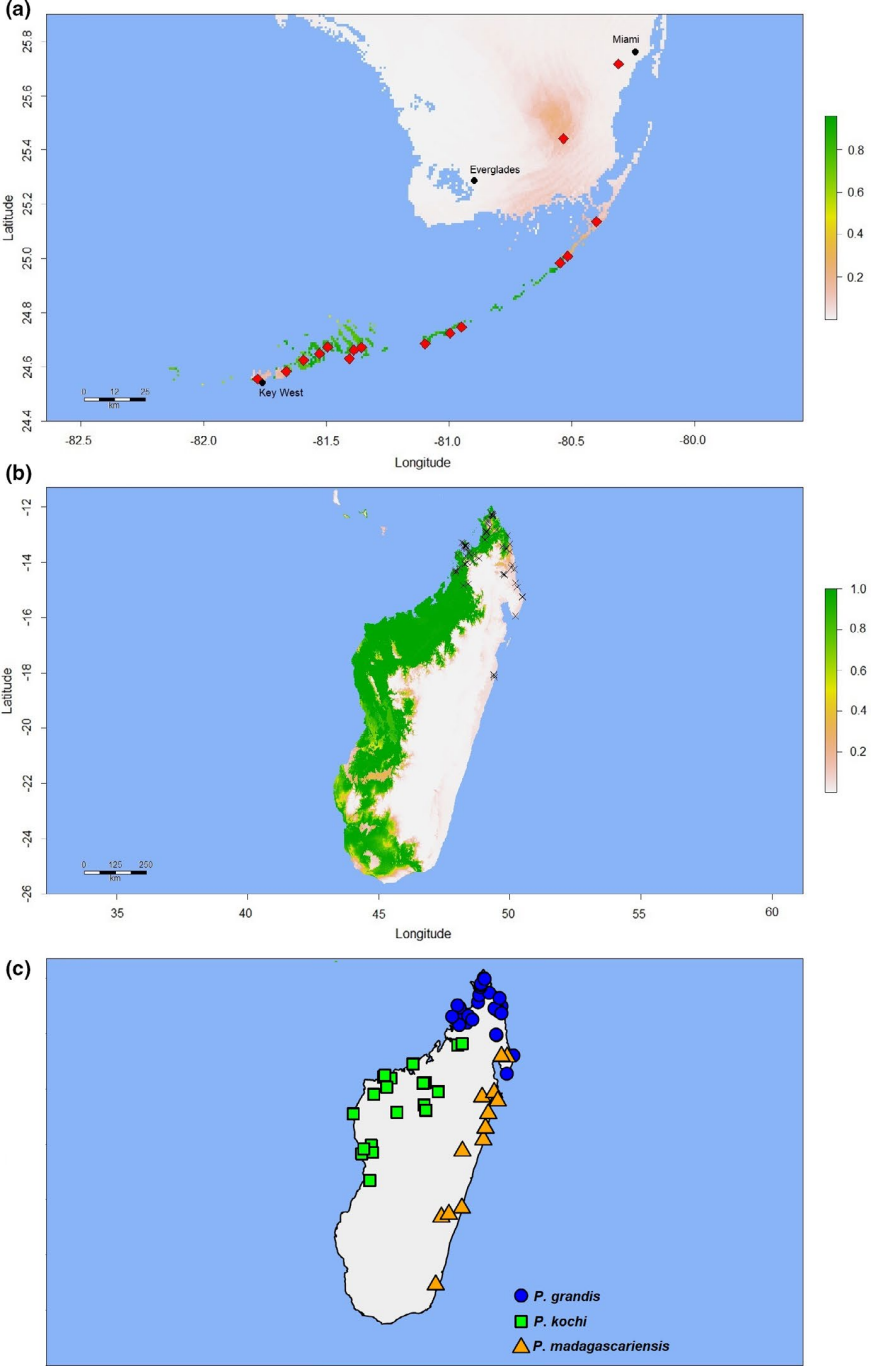
“Florida” Maxent model built using Florida presence data and showing predicted habitat suitability for *Phelsuma grandis* in (a) southern Florida and (b) its native Madagascar. Diamonds denote the approximate locations of known Florida *P*. *grandis* populations; crosses denote georeferenced *P*. *grandis* observations from Madagascar. The scale bars to the right of the maps indicate the degree of predicted habitat suitability, with higher scores representing predicted higher suitability, with the range of possible values 0–1. (c) Presence records of *Phelsuma grandis* (circles), *P*. *kochi* (squares), and *P*. *madagascariensis* (triangles) in Madagascar; presence data shown are taken from the Supplementary data of C. J. Raxworthy et al. (2007). Note that (c) is not a model projection and is meant only to illustrate the native range of the *Phelsuma* species of interest

The Florida model projection (Figure 5a) closely resembles the known distribution of *P*. *grandis* in southern Florida (Fieldsend & Krysko, 2019b). Predicted suitability values for the 24 Florida validation points were between 0.109 and 0.939 (mean = 0.692; median = 0.838). Temperature Seasonality (BIO4) accounted for 69.1% of the permutation importance of the Florida model, while Mean Temperature of the Driest Quarter (BIO9) accounted for 18.9%. When projected onto Madagascar (Figure 5b), the Florida model‐predicted distribution was not in accordance with the native distribution of *P*. *grandis*, with substantial erroneous additional distribution predicted in parts of western Madagascar occupied by *P*. *kochi*, and in areas of southwestern and southern Madagascar occupied by neither species (Figure 5c). P10 omission error rate for native‐range presence points was 31% (19/62). Predicted suitability values for the 62 native‐range presence points ranged from 0.0009 to 1 (mean = 0.620; median = 0.894), and the relatively high mean and median values are probably partially an artifact of the large swath of Madagascar predicted to be suitable for *P*. *grandis* by the Florida model. Nevertheless, the mean predicted suitability value for the 62 native‐range presences was significantly higher than that of 62 points randomly generated within the same spatial extent as the model projection (0.620 vs. 0.423, Student's paired *t* test *p* < .005, data not shown), implying some degree of predictive power.

**FIGURE 6:**
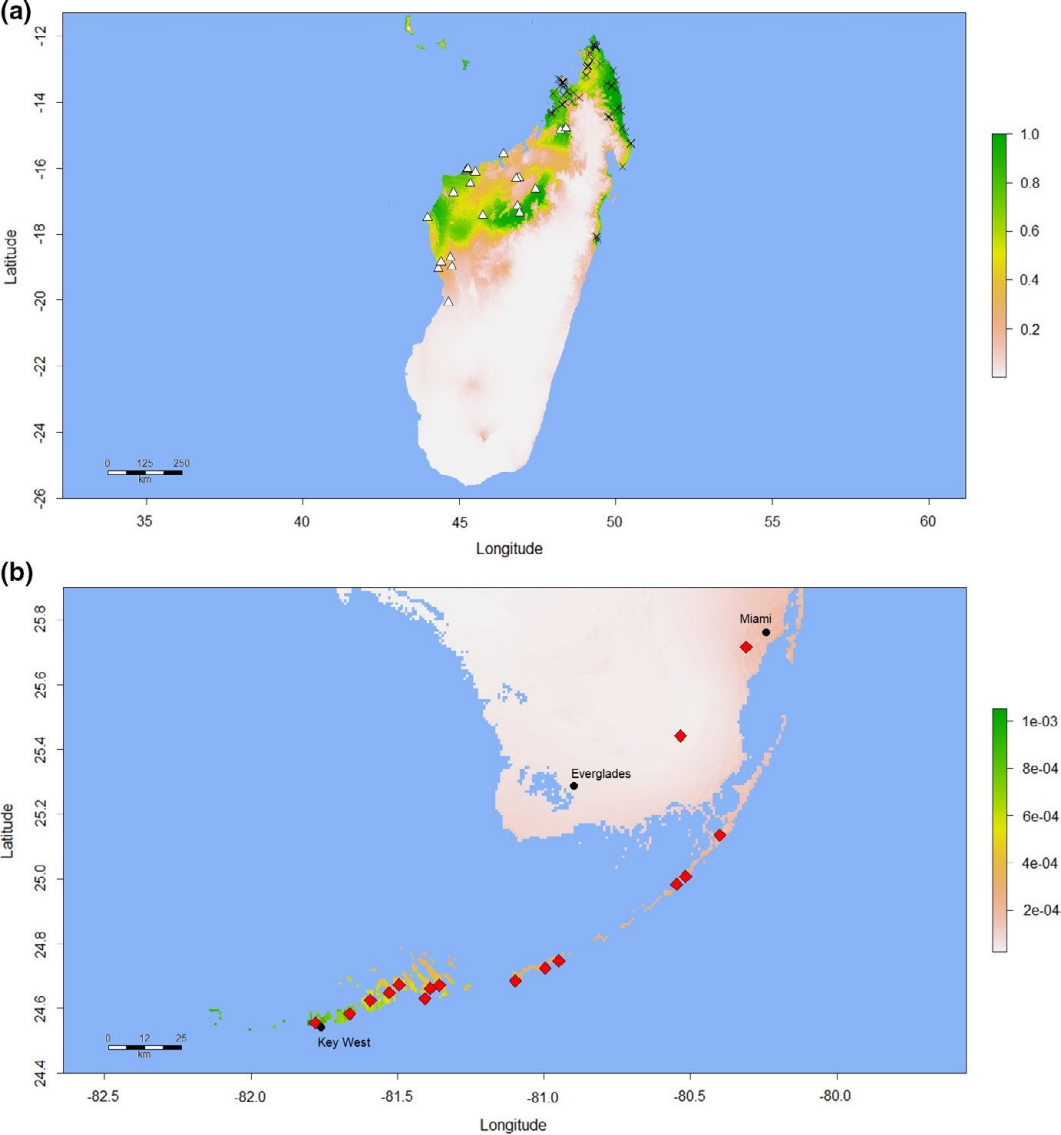
“*kochi*/*grandis*” Maxent model built using combined native‐range presence data for *Phelsuma grandis* and *P*. *kochi* and showing predicted habitat suitability in (a) Madagascar and (b) southern Florida. The georeferenced *P*. *grandis* (*n* = 62) and *P*. *kochi* (*n* = 21) observations used to construct the model are denoted by crosses (*P*. *grandis*) and triangles (*P*. *kochi*); diamonds denote the approximate locations of known Florida *P*. *grandis* populations. The scale bars to the right of the maps indicate the degree of predicted habitat suitability, with higher scores representing predicted higher suitability, with the range of possible values 0–1

The *kochi*/*grandis* model projection (Figure 6a) broadly describes the combined native range of *P*. *grandis* and *P*. *kochi* (Figure 5c), albeit with some modest underprediction evident in some areas of the range occupied by *P*. *kochi*. Predicted suitability values for the native‐range validation points (*n* = 29) were between 0.038 and 1 (mean = 0.673; median = 0.742). The variable of highest permutation importance was Temperature Seasonality (BIO4; 43.8%), followed by Minimum Temperature of the Coldest Month (BIO6; 19.5%) and Isothermality (BIO3; 15%). The projection of the *kochi/grandis* model onto southern Florida (Figure 6b) was in agreement with the Madagascar and Florida models in identifying the Florida Keys as some of the most bioclimatically suitable area within Florida; however, none of Florida was characterized as bioclimatically suitable habitat in absolute terms (P10 = 0.427; omission error rate = 100%). Predicted suitability values for the 70 *P*. *grandis* Florida presence points were low—ranging from 0.00003 to 0.001 (mean = 0.0004; median = 0.0004)—but were significantly higher on average than that predicted by the Madagascar model (0.0004 vs. 0.00003, Student's paired *t* test *p* < .001, data not shown). Nevertheless, when validated using the 70 Florida *P*. *grandis* presence points, both models displayed high discriminatory capacity (Madagascar model AUC 0.982; *kochi*/*grandis* model AUC 0.947) despite the low average‐predicted suitability values for presence records.

## 4 DISCUSSION

Our study tested the predictive accuracy of the widely advocated climate‐matching species distribution modeling approach by using Maxent, *Bioclim* variables, and native‐range presence‐only data to identify areas of potential bioclimatic suitability for *Phelsuma grandis* in Florida, USA, and then comparing these predictions with the species’ known distribution in the state. Interestingly, our model did not identify any of the already‐colonized habitat as potentially suitable for *P*. *grandis*, demonstrating that climate‐matching SDMs can severely underpredict the establishment risk posed by non‐native herpetofauna.

It seems probable that the discrepancy between the predicted and observed distribution stems from the inherent assumptions of many SDMs, namely that 1) the observed native range of a taxon represents its fundamental bioclimatic niche and 2) adaptation to novel bioclimatic conditions will be trivial or nonexistent (Dormann, 2007; Uden et al., 2015). While these assumptions hold true to an extent for groups such as terrestrial plants (Petitpierre et al., 2012), they demonstrably do not for many non‐native reptiles: Li et al. (2014) showed that 61% of the 46 non‐native reptiles that they studied inhabited novel bioclimatic conditions in their non‐native range, while the equivalent figure in a similar study by Wiens et al. (2019) was 58% (36/52). This phenomenon can occur because a species’ native range represents only a subset of its fundamental niche (Wiens et al., 2019), and other factors—such as biological interactions and dispersal limitations—hinder its further spread (Li et al., 2014).

Non‐native species often respond to novel environments via rapid in situ adaptation (Angetter et al., 2011; Kolbe et al., 2013; Stroud et al., 2020). Such in situ adaptation can be both behavioral (e.g., changing behavioral thermoregulation patterns; Brown, 1996; Lapwong et al., 2020) and physiological (e.g., increased cold tolerance; Kolbe et al., 2012; Leal & Gunderson, 2012; Stroud et al., 2020) and has been documented in a number of non‐native reptiles (Kolbe et al., 2012, 2013; Lapwong et al., 2020; Leal & Gunderson, 2012; Stroud et al., 2020; While et al., 2015), including geckos (Lapwong et al., 2020; Stroud et al., 2020) and non‐native lizards in Florida (Kolbe et al., 2012, 2013; Leal & Gunderson, 2012; Stroud et al., 2020).

**FIGURE 7:**
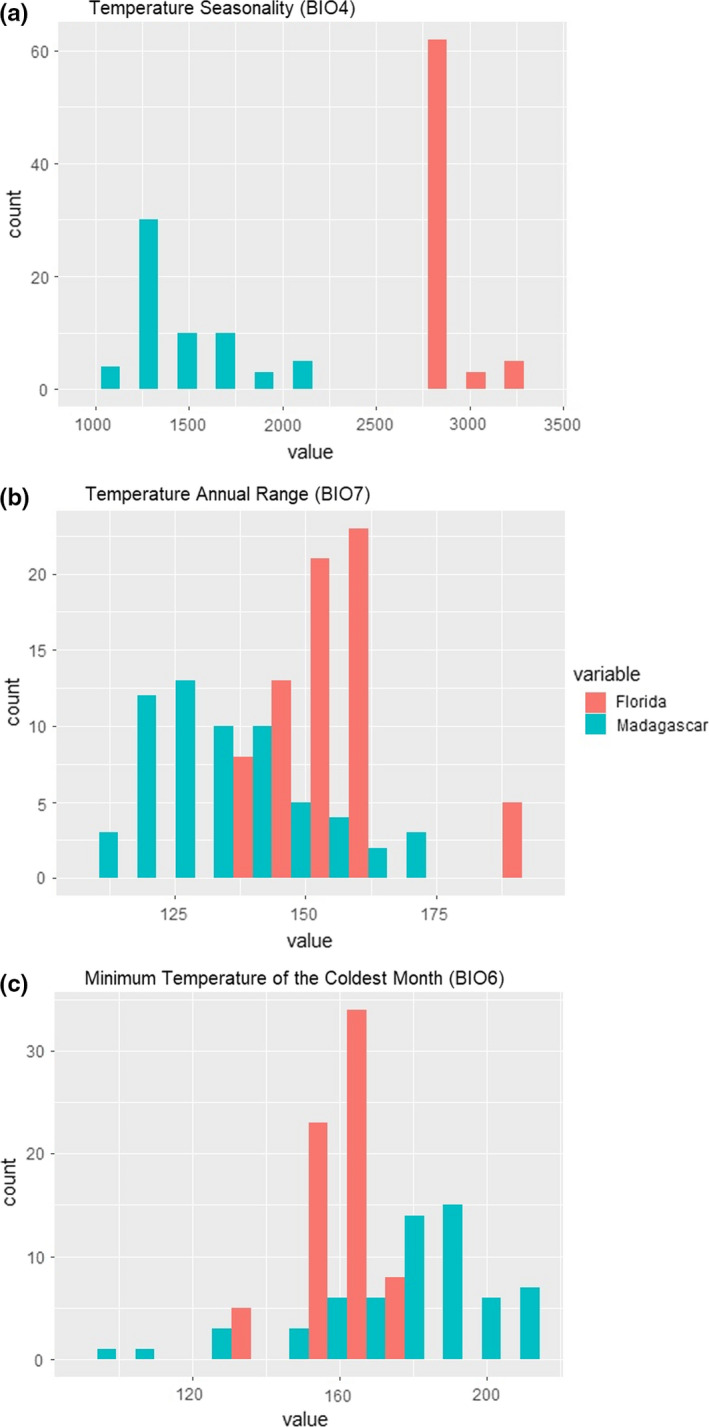
Histograms showing the *Bioclim* values associated with *Phelsuma grandis* presence points from Madagascar (turquoise) (*n* = 62) and Florida (red) (*n* = 70). (a) Temperature Seasonality (BIO4), a measure of temperature change over the course of a year calculated using the standard deviation of monthly temperature averages, with higher values corresponding to greater seasonal variability in temperature; (b) Temperature Annual Range (BIO7), calculated by subtracting the minimum temperature of the coldest month from the maximum temperature of the warmest month, with higher values corresponding to greater annual ranges in temperature; and (c) Minimum Temperature of the Coldest Month (BIO6), with higher values corresponding to higher minimum temperatures

The extent to which bioclimatic factors truly constrain the distribution of *P*. *grandis* in Florida remains unclear. Given its tropical native‐range distribution (Raxworthy et al., 2007), a reasonable a priori assumption would have been that the range of *P*. *grandis* in Florida was primarily limited by temperature, especially as the species has yet to successfully establish itself beyond the tropical southern tip of the state (Fieldsend & Krysko, 2019b). Indeed, Temperature Seasonality had a permutation importance of around 70% for both the Madagascar and Florida models, with Madagascar locales found to be associated with less seasonal thermal variability than those in Florida (Figure 7a). Curiously, however, the Temperature Annual Range comparison was quite equivocal (Figure 7b), and Minimum Temperature of the Coldest Month (BIO6) (Figure 7c) had a permutation importance of 0% for both the Madagascar and Florida models, suggesting that extreme cold‐weather events might not be the primary limiting factor for *P*. *grandis* in either Madagascar or Florida. While *Phelsuma grandis* has been documented withstanding temperatures as low as −1.1°C (30°F) in Homestead, Florida (Fieldsend & Krysko, 2019a), comparative analysis reveals that a handful of native‐range *P*. *grandis* observations are actually associated with even more extreme values for Minimum Temperature of the Coldest Month than the Homestead location (Figure 7c).

The lack of evolutionary conservatism in the critical thermal minima (CT_min_) (Brown, 1996) of lizards (Grigg & Buckley, 2013) suggests that *P*. *grandis* could potentially adapt physiologically to colder ambient temperatures, for instance if dispersing northward through Florida. Rapid in situ physiological adaptation of this nature has already been reported for several non‐native lizard species in Florida (Stroud et al., 2020). Furthermore, since its initial establishment in Florida in the 1990s, *P*. *grandis* has been exposed to extreme cold‐weather events that have caused substantial cold‐induced mortality in multiple non‐native squamate species (Campbell, 2011; Fieldsend & Krysko, 2019a; Mazzotti et al., 2011, 2016), illustrating how powerful selective forces might drive rapid population‐level adaptation. Unlike most geckos, *P*. *grandis* is diurnal (Dubos, 2013), thereby allowing it greater scope for behavioral thermoregulation than nocturnal gekkotans (Brown, 1996). The species is also synanthropic (D'Cruze et al., 2009; Dubos, 2013; Dubos et al., 2014; Krysko et al., 2003), and so likely benefits from both the urban heat island effect (Campbell‐Staton et al., 2020) and access to the warmer microhabitats associated with some anthropogenic structures (Hulbert et al., 2020; Lapwong et al., 2020; Sievert & Hutchison, 1988), which may also help to explain the low predictive power of minimum temperature. It is thus possible that both behavioral and physiological adaptations contribute to the observed ability of *P*. *grandis* to endure brief periods of extreme cold in southern Florida (Fieldsend & Krysko, 2019a). Given its tropical native range, it seems likely that the intensity and frequency of extreme cold events must ultimately limit the northward expansion of *P*. *grandis* (e.g., Warner et al., 2021). However, the high permutation importance of Temperature Seasonality—along with the lack of overlap in values for Temperature Seasonality between the Madagascar and Florida ranges (Figure 7a)—suggests that exposure to extended periods of suboptimal temperatures probably also plays an important limiting role (e.g., Battles & Kolbe, 2019; Nania et al., 2020).

In Madagascar, the range of *P*. *grandis* is parapatric with the distribution of the closely related species *P*. *kochi* and *P*. *madagascariensis*, with little or no spatial overlap (Raxworthy et al., 2007). The projection of the Florida model onto Madagascar prima facie suggests that the distribution of *P*. *grandis* in Madagascar could be limited by the presence of *P*. *kochi*, which is acting as a competitor, and thus excluding *P*. *grandis* from occupying areas within its fundamental niche (as identified by the Florida model) in western, southwestern, and southern Madagascar (Figure 5b,c). There are no obvious geographic barriers that are otherwise preventing *P*. *grandis* from occupying these areas, and the extreme ecological flexibility exhibited by *P*. *grandis* in both northern Madagascar and southern Florida suggests that it should also flourish in the primary forests and human‐degraded habitats of western Madagascar. While *P*. *kochi* does not occur in southwestern and southern Madagascar (Figure 5c), our model implies that—saltatory dispersal notwithstanding (e.g., Deso, 2001; Dubos et al., 2014)—*P*. *grandis* would have to disperse through *P*. *kochi*‐occupied habitat in western Madagascar to reach suitable habitat in these regions (Figure 5b,c), perhaps explaining its current absence. Another interesting implication of our model is that Florida *P*. *grandis*—but not necessarily Madagascar *P*. *grandis*—are potentially capable of colonizing parts of southern and southwestern Madagascar known to harbor high numbers of endemic and threatened species (D’Cruze et al., 2009). A “secondary introduction” (Kolbe et al., 2004) of Florida *P*. *grandis* to this region of Madagascar could thus be highly destructive, given the impact that introduced *P*. *grandis* populations can have on endemic species (Buckland et al., 2014; Sanchez & Probst, 2014). Since many *P*. *grandis* in the United States pet trade are in fact “harvested” from wild Florida populations (Krysko et al., 2019), such a scenario is plausible if export of captive *P*. *grandis* from the USA to Madagascar or the Mascarene Islands occurs.

Overlapping fundamental niches of recently speciated sister species is a prediction of ecological speciation on environmental gradients (ecotones), and the *Phelsuma madagascariensis* complex—comprising *P*. *grandis*, *P*. *kochi*, and *P*. *madagascariensis*—has been considered as a strong candidate for ecological speciation (Raxworthy et al., 2007). Interestingly, the Florida model—when projected onto Madagascar—provides no evidence for extensive overlap in the fundamental niches of *P*. *grandis* and *P*. *madagascariensis* (Figure 5b,c). Despite the morphological similarity of all three species, *P*. *madagascariensis* is more distantly related to *P*. *kochi* and *P*. *grandis* than they are to one another (Rocha et al., 2010) and may have evolved a fundamental niche quite distinct from that of either *P*. *grandis* or *P*. *kochi*. If true, this would further support the claim that ecological speciation has occurred within this species complex. However, a lack of climate analogues between Florida and the native range of *P*. *madagascariensis* could also lead to a similar prediction; in this case, *P*. *grandis* would by definition be unable to establish in such areas in Florida, and model output would consequently be biased against them.

Our results provide some evidence that the colonization of Florida by *P*. *grandis* may have been facilitated by ecological release (Kohn, 1972), in this case, from interspecific competition with *P*. *kochi*. However, given that the projection of the Florida model onto Madagascar (Figure 5b) identifies more suitable native‐range habitat for *P*. *grandis* than is identified even by the combined‐species *kochi*/*grandis* model (Figure 6a), we suggest that some degree of in situ adaptation has almost certainly occurred during this colonization event, as a result of which the *P*. *grandis* population of Florida has expanded its occupied niche. Since the *kochi*/*grandis* model did not predict the observed successful colonization of Florida by *P*. *grandis* with high accuracy (Figure 6b), the degree to which competition with *P*. *kochi* restricts the distribution of *P*. *grandis* to northern Madagascar—and thus prevents the latter from occupying a portion of its fundamental niche—remains an open question. The likelihood of ecological release could be tested in semi‐natural experimental enclosures in Madagascar containing *P*. *grandis* and *P*. *kochi* (sensu Wright, 2019), in order to determine the extent to which *P*. *kochi* truly outcompetes *P*. *grandis*. Moreover, experimental proof that Florida *P*. *grandis* are significantly more cold‐tolerant (e.g., lower CT_min_; Kolbe et al., 2012) than *P*. *grandis* from far northern Madagascar—the probable source region of most Florida *P*. *grandis* (Fieldsend et al., 2021)—would be very strong evidence that in situ adaptation has indeed occurred. Knowledge of the factors underlying the discrepancy between the predicted and observed distribution of *P*. *grandis* in Florida would inform the management of similar species currently colonizing Florida, such as *P*. *laticauda* (Fieldsend et al., 2020).

In summary, our study adds weight to the argument that climate‐matching SDMs generated from native‐range distributional data may not alone be appropriate tools for predicting the establishment risk of non‐native herpetofauna (Li et al., 2014). In particular, our results highlight an example of an invasive species whose occupied native‐range niche is much smaller than its non‐native‐range niche, due to in situ adaptation in the non‐native range, and potentially also competition with a closely related species within the native range. We suggest that modeling approaches accounting for dispersal and biotic interactions (e.g., Boulangeat et al., 2012), incorporating relevant behavioral/physiological data (i.e., "mechanistic" models, e.g., Stahl et al., 2016), and utilizing fine‐scale habitat data (e.g., Mutascio et al., 2018) show promise as tools for managing non‐native herpetofauna, and can further build upon insights gained from climate‐matching approaches.

## CONFLICT OF INTEREST

The authors state that there is no conflict of interest.

## AUTHOR CONTRIBUTIONS

**Thomas W. Fieldsend:** Conceptualization (lead); Data curation (equal); Formal analysis (lead); Funding acquisition (supporting); Investigation (lead); Methodology (lead); Project administration (lead); Resources (equal); Software (equal); Supervision (supporting); Validation (equal); Visualization (equal); Writing‐original draft (lead); Writing‐review & editing (lead). **Nicolas Dubos:** Conceptualization (equal); Data curation (equal); Formal analysis (equal); Funding acquisition (supporting); Investigation (equal); Methodology (equal); Project administration (equal); Resources (equal); Software (equal); Supervision (equal); Validation (equal); Visualization (equal); Writing‐original draft (supporting); Writing‐review & editing (supporting). **Kenneth L. Krysko:** Conceptualization (equal); Data curation (equal); Formal analysis (supporting); Funding acquisition (supporting); Investigation (equal); Methodology (supporting); Project administration (equal); Resources (equal); Software (supporting); Supervision (equal); Validation (equal); Visualization (equal); Writing‐original draft (supporting); Writing‐review & editing (supporting). **Christopher J. Raxworthy:** Conceptualization (equal); Data curation (equal); Formal analysis (supporting); Funding acquisition (lead); Investigation (equal); Methodology (equal); Project administration (equal); Resources (equal); Software (supporting); Supervision (equal); Validation (equal); Visualization (equal); Writing‐original draft (supporting); Writing‐review & editing (supporting). **Sparkle L. Malone:** Conceptualization (equal); Data curation (equal); Formal analysis (equal); Funding acquisition (lead); Investigation (equal); Methodology (equal); Project administration (equal); Resources (equal); Software (equal); Supervision (lead); Validation (equal); Visualization (equal); Writing‐original draft (supporting); Writing‐review & editing (supporting).

## Supporting information

Appendix S1

Appendix S2

Appendix S3

Appendix S4

Appendix S5

## Data Availability

A ZIP file containing (a) the datafiles used in this study, (b) the R script used to generate, project, and analyze the models presented in this paper, and (c) the output files associated with the models is available via Dryad, DOI https://doi.org/10.5061/dryad.m905qfv1c.
